# Hepatitis B Virus: Inactive carriers

**DOI:** 10.1186/1743-422X-2-82

**Published:** 2005-09-28

**Authors:** Sanjeev Kumar Sharma, Nitin Saini, Yogesh Chwla

**Affiliations:** 1 Head, Department of Hepatology, PGIMER, Chandigarh, 160012 India

## Abstract

Inactive carriers forms the largest group in chronic HBV infected patients. Around 300 million people are inactive carriers The inactive HBsAg carrier state is diagnosed by absence of HBeAg and presence of anti-HBe, undetectable or low levels of HBV DNA in PCR-based assays, repeatedly normal ALT levels, and minimal or no necroinflammation, slight fibrosis, or even normal histology on biopsy. Inactive cirrhosis may be present in patients who had active liver disease during the replicative phase of infection. The prognosis of the inactive HBsAg carrier state is usually benign. Long-term follow- up (up to 18 years) of these carriers has indicated that the vast majority show sustained biochemical remission and very low risk of cirrhosis or hepatocellular carcinoma (HCC). Rarely, patients, even noncirrhotics, may develop liver cancer during the inactive HBsAg carrier state. In addition, approximately 20 to 30% of persons in the inactive HBsAg carrier state may undergo spontaneous reactivation of hepatitis B during follow-up. Multiple episodes of reactivation or sustained reactivation can cause progressive hepatic damage and even hepatic decompensation. Introduction

## 

Hepatitis B virus (HBV) infection and its sequelae are major global health problems [1]. It is estimated that 400 million people worldwide are HBV carriers [2]. The natural history of hepatitis B is complex and is influenced by many factors, including age at infection, viral factors (HBV genotype, viral mutations, level of HBV replication), host factors (gender, age, and immune status), and exogenous factors such as concurrent infection with other hepatotropic viruses or alcohol. The clinical spectrum of HBV infection ranges from subclinical to acute symptomatic hepatitis or, rarely, fulminant hepatitis during the acute phase and from the inactive hepatitis B surface antigen (HBsAg) carrier state to chronic hepatitis, cirrhosis, and its complications during the chronic phase [3,4]. Approximately 15 to 40% of people who develop chronic HBV infection are expected to progress to cirrhosis and end-stage liver disease [1]. Difficulties in defining the natural history of chronic hepatitis B include the indolent course of the disease, the lack of symptoms during the early stages, and the heterogeneity of the disease. Understanding the natural history and prognosis of hepatitis B is the basis for disease management and for designing better therapeutic strategies.

## Hepatitis B Virus

HBV belongs to the family hepdnaviruses. The HBV genome is a relaxed circular, partially double stranded DNA of approximately 3,200 base pairs. There are four partially overlapping open reading frames encoding the envelope (pre-S/S), core (precore/core), polymerase, and X proteins [5]. The pre-S/S open reading frame encodes the large, middle, and small surface glycoproteins. The precore/core open reading frame is translated into precore polypeptide which is modified in to a soluble protein, the hepatitis B e antigen (HBeAg), and the nucleocapsid core protein hepatitis B core antigen (HBc Ag) [5]. The polymerase protein functions as reverse transcriptase as well as DNA polymerase. The X protein is a potent transactivator and may play role in hepatocarcinogenesis.

## Prevalence

Hepatitis B is spread predominantly parenterally, through intimate personal contact, and perinatally. Individuals at risk are intravenous drug users, children of mothers with HBV, men who have sex with men, patients on hemodialysis and those exposed to blood or blood products [6,7]. Approximately 5% of the world's populations are carriers of HBV, defined as being positive for hepatitis B surface antigen. HBV is endemic in many areas of the world, such as Asia, Micronesia, and sub-Saharan Africa as well as in certain populations in Australia, New Zealand, South America, the Middle East and the Arctic. An estimated 1.25 million people in the United States are positive for hepatitis B surface antigen. Fifteen percent to forty percent of these carriers may develop hepatitis B-related sequelae in their lifetimes [8-10].

## Natural History

Perinatal infection of infants from infected mothers and horizontal infection early in childhood from exposure to HBsAg-positive family members are the main routes of HBV transmission in highly endemic areas, such as Southeast Asia, Africa, the Pacific Islands, and the Arctic. In regions of low endemicity, such as Western countries, hepatitis B is primarily a disease of adolescents and adults as a result of high-risk sexual behavior and injection drug use. HBV infection is a dynamic process with replicative and nonreplicative (or low replicative) phases based on virus-host interaction. The presence of circulating HBsAg, hepatitis B e antigen (HBeAg), and high levels of serum HBV DNA characterizes the immunotolerant phase. This first phase is seen in patients with perinatal infection and often lasts for decades. During this phase patients have no symptoms, normal or slightly increased serum alanine aminotransferase (ALT) levels, and minimal histological activities, which imply that there is a lack of or a very weak immune response against the infected hepatocytes.

Experimental results in transgenic mice suggested HBeAg induces a state of immunological tolerance to HBV in neonates [11]. During the course of chronic HBV infection, for unknown reasons, the tolerogenic effect is and patients may enter the immunoactive phase, which is associated with a decrease in HBV DNA concentrations and increased ALT levels and histologic activity, reflecting immune-mediated lysis of infected hepatocytes. This second phase has a variable duration from months to years. The third low or nonreplicative phase occurs seroconversion from HBeAg to antibody to HBeAg. This phase is usually preceded by a marked reduction of serum HBV DNA to levels that are not detectable by hybridization techniques, followed by normalization of ALT levels and resolution of liver necroinflammation. In many patients, serum HBV DNA remains detectable by the sensitive technique of polymerase chain reaction (PCR). This phase is also referred as the inactive HBsAg carrier state [3,4]. The inactive carrier state may for a lifetime, but a proportion of patients may undergo subsequent spontaneous or immunosuppressioninduced reactivation of HBV replication with reappearance of high levels of HBV DNA with or without HBeAg seroreversion and a rise in ALT levels [3]. For reasons that are not yet known, replication-competent HBV variants with mutations in the precore or core promoter regions preventing or down-regulating HBeAg production may be selected during or after HBeAg seroconversion.

Patients who become HBsAg negative and develop antibody to HBsAg (anti-HBs) are diagnosed as having resolved hepatitis B [3,4]. This is an uncommon phenomenon in chronic HBV infection. During stage HBV DNA may still be detectable by PCR in serum and more often in the liver.[12] In rare cases severe immune suppression, such as cancer chemotherapy or after organ transplantation, HBV can be reactivated in patients with resolved hepatitis B [13].

## Clinical spectrum

### HBeAg positive Chronic Hepatitis

Patients with HBeAg-positive chronic hepatitis B usually present in the third or fourth decade of life. Men outnumber women, [14,15] liver damage ranges from mild (24 to 42%) to moderate or severe chronic hepatitis (44 to 63%) or active cirrhosis (10 to 24%) [16-20]. Chronic hepatitis B tends to be milder in children. Nevertheless, severe liver disease including cirrhosis may occur in a small proportion of patients during childhood [21,22]. A key event in the natural history of HBeAg positive chronic hepatitis is HBeAg seroconversion. Several studies have shown that seroconversion with marked reduction of HBV replication is associated with biochemical and histologic remission of inflammatory activity in the majority of patients [23-25]. Regression of fibrosis occurs gradually months to years after HBeAg seroconversion.[26] In longitudinal studies the observed probability of clearing HBeAg was about 50% and 70% within 5 and 10 years of diagnosis, respectively [23,27-29]. Most studies have found that the mean annual rate of spontaneous HBeAg seroconversion ranges from 8 to 15% in children or adults with elevated ALT [3,4,23-25,29-31,31]. Among Asian, most of whom have normal ALT, spontaneous HBeAg seroconversion occurs at a very low rate, less than 2% during the first 3 years of age and 4 to 5% in children older than 3 years [32]. Several determinants for HBeAg seroconversion have been reported, including gender, age, ALT level, and more recently HBV genotypes. Older carriers and females are more likely to clear HBeAg [33].

Frequent acute exacerbation of hepatitis, reflecting immune-mediated lysis of HBV-infected hepatocytes with ALT elevations to more than 10 times ULN and more than twice the baseline value and with HBV DNA levels rising before and falling during the flare, precede seroconversion of HBeAg to anti-HBe. These exacerbations usually last 2 to 4 months [34]. In some cases these spontaneous flares of hepatitis are not followed by subsequent HBeAg seroconversion and can be viewed as an abortive attempt at seroconversion. These flares of hepatitis are usually asymptomatic and frequently unrecognized, but some are accompanied by symptoms of acute hepatitis and rarely, primarily in patients with cirrhosis or advanced fibrosis, may lead to hepatic decompensation and even death due to massive necrosis [34].

### HBsAg-negative chronic hepatitis

The diagnosis of HBeAg-negative chronic hepatitis B is based on the presence of HBsAg for more than 6 months, undetectable HBeAg, presence of anti-HBe, detectable serum HBV DNA exceeding 10^5 ^to 10^6 ^copies/mL, increased ALT levels, and hepatic necroinflammation on histology. Other causes of liver disease, such as superinfection with other hepatitis viruses, alcohol abuse, hepatotoxic drug use, and autoimmune or metabolic liver disease, should be excluded [3,4]. The atypical serological profile is related to the predominance of HBV variants, which are unable to express HBeAg. The most frequent variant has a G-to-A change at nucleotide 1896 (G1896A), which creates a stop codon in the precore region of the HBV genome and completely abolishes the production of HBeAg [35]. Other variants include changes in the start codon of the precore region or a two-nucleotide substitution (A1762T, G1764A) in the core promoter region, which reduces precore messenger RNA synthesis and HBeAg production [36].

Patient with HBeAg negative are older than patients with HBeAg-positive chronic hepatitis (median 40, range 36–45 years). Males predominate and data indicate that liver disease is more active and advanced, minimal or mild chronic hepatitis is infrequent, and severe necroinflammation is seen in more than 50% patients at diagnosis [37-39]. In reports from Mediterranean area, 29 to 38% had cirrhosis at presentation. The older age and the high rate of advanced liver damage at presentation suggest that HBeAg-negative chronic hepatitis represents a late phase in the natural history of chronic HBV infection rather than de novo infection with HBV variants that do not produce HBeAg. To further support this concept, a recent long-term study reported that HBeAg-negative chronic hepatitis accumulated over time after HBeAg seroconversion with a cumulative incidence of approximately 25% after 16 years of follow-up [40]. Thus, the increasing prevalence of HBeAg-negative. Fluctuation in level of viremia and ALT are more common and sustained response is rare. Delayed spontaneous HBsAg clearance has been estimated to occur at a low rate of 0.5% per year [40].

### Inactive HBsAg Carrier State

Inactive carriers forms the largest group in chronic HBV infected patients. Around 300 million people are inactive carriers The inactive HBsAg carrier state is diagnosed by absence of HBeAg and presence of anti-HBe, undetectable or low levels of HBV DNA in PCR-based assays, repeatedly normal ALT levels, and minimal or no necroinflammation, slight fibrosis, or even normal histology on biopsy [3,4]. Inactive cirrhosis may be present in patients who had active liver disease during the replicative phase of infection. The prognosis of the inactive HBsAg carrier state is usually benign. Long-term follow- up (up to 18 years) of these carriers has indicated that the vast majority show sustained biochemical remission and very low risk of cirrhosis or hepatocellular carcinoma (HCC) [40-42]. Rarely, patients, even noncirrhotics, may develop liver cancer during the inactive HBsAg carrier state [40-43]. In addition, approximately 20 to 30% of persons in the inactive HBsAg carrier state may undergo spontaneous reactivation of hepatitis B during follow-up [29,33,34,40]. Multiple episodes of reactivation or sustained reactivation can cause progressive hepatic damage and even hepatic decompensation. HBV reactivation is usually asymptomatic but on occasion can mimic acute viral hepatitis [44]. Acute flares of hepatitis should be differentiated from superinfection with other hepatotropic viruses. As many as 20 to 30% of these acute exacerbations may be caused by superinfection with HDV, HCV, or hepatitis A virus and can be associated with an increased risk of fulminant hepatic failure [44]. Some carriers eventually become HBsAg negative and develop anti-HBs. The incidence of delayed HBsAg clearance has been estimated to be 1 to 2% per year in Western countries, where HBV infection is usually acquired in adulthood, but a lower rate from 0.05 to 0.8% per year in endemic areas, where HBV infection is mostly acquired perinatally or in early childhood. Clearance of HBsAg has been reported to be higher in women than in men and in older than younger carriers. Prognosis is improved by loss of HBsAg as liver disease is usually inactive and nonprogressive, but HBsAg clearance does not completely prevent occurrence of decompensation or HCC in patients who have already developed cirrhosis [45,46].

### Change in the terminology of HBV carriers

HBV infection is termed as chronic if it continues to be HBsAg +ve for ≥6 months. Chronic HBV infection is a dynamic process with a wide spectrum of spectrum of affliction. On one hand patients are asymptomatic with no clinical evidence of liver diseases, while on other being end-stage cirrhosis and hepatocellular carcinoma. For many decades the patients were considered to have a benign, non progression infection and were designated as hepatitis B "carriers". Probably the word 'carrier' was mistakenly chosen for hepatitis B as in true sense, a carrier is an individual who (i) harbors a specific infectious agent (ii) has no discernible clinical disease and (iii) serves as a potential source of infection. For this infection the second and third points should be looked at carefully. One the basis of Asian collaborative survey the term 'carrier' was replaced by the term 'chronic hepatitis B virus infection' [47,48]. Later on for this infection the term 'Inactive HBsAg carrier' was adopted [49].

## Management of Inactive HBsAg Carrier

Differentiation from chronic HBsAg negative hepatitis B, requires serial testing of ALT and HBV DNA for one year before designating carrier state [49]. In subject with inactive carrier state testing of HBV DNA and liver biopsy are not recommended. Treatment is not recommended as there is no evidence that available therapy affects HBsAg status. Family screening with HBsAg and anti-HBs, if negative vaccinate them and success of vaccination should be confirmed with anti-HBs testing. Protected sexual intercourse until partner has developed protective antibodies. The offspring need active and passive vaccination [4,47]. Use of alcohol should be avoided, possibility of reactivation or super infection by other viruses and advised if there is jaundice, malaise or increased fatigue. Regular follow-up at every 6–12 months intervals with ALT [4]. If the age of the patient is more than 50 yrs family history of HCC-AFP and ultrasonography every 6–12 monthly should be done. Universal precaution should be taken while treating these patients in the hospital. They should not be allowed to donate the blood or organ or semen. For pregnant women vaccinate the new born at birth with active and passive immunization with in 12 hours of the birth, close monitoring required if undergoing chemotherapy or immunosuppressive medication.
